# A multi-region single nucleus transcriptomic atlas of Parkinson’s disease

**DOI:** 10.1038/s41597-024-04117-y

**Published:** 2024-11-23

**Authors:** Prashant N. M., John F. Fullard, Tereza Clarence, Deepika Mathur, Clara Casey, Evelyn Hennigan, Marcela Alvia, Joana Krause-Massaguer, Ayled Barreda, David A. Davis, Regina T. Vontell, Susanna P. Garamszegi, Jeffery M. Vance, Lorelle Sang, Michael Chatigny, David Vismer, Barry Landin, David Burstein, Donghoon Lee, Georgios Voloudakis, Sabina Berretta, Vahram Haroutunian, William K. Scott, Jaroslav Bendl, Panos Roussos

**Affiliations:** 1 Center for Disease Neurogenomics, Icahn School of Medicine at Mount Sinai, New York, NY USA; 2 Friedman Brain Institute, Icahn School of Medicine at Mount Sinai, New York, NY USA; 3 Department of Psychiatry, Icahn School of Medicine at Mount Sinai, New York, NY USA; 4 Department of Genetics and Genomic Sciences, Icahn School of Medicine at Mount Sinai, New York, NY USA; 5 Mental Illness Research Education and Clinical Center (VISN 2 South), James J. Peters VA Medical Center, Bronx, NY USA; 6 Center for Precision Medicine and Translational Therapeutics, James J. Peters VA Medical Center, Bronx, NY USA; 7 Brain Endowment Bank, Department of Neurology, University of Miami Miller School of Medicine, Miami, FL USA; 8 John P. Hussman Institute for Human Genomics, University of Miami Miller School of Medicine, Miami, FL USA; 9 McLean Hospital, Belmont, MA USA; 10 Technome LLC, Herndon, VA USA; 11 Stanley Center for Psychiatric Research, Broad Institute of MIT and Harvard, Cambridge, MA USA; 12 Department of Psychiatry, Harvard Medical School, Boston, MA USA; 13 Department of Neuroscience, Icahn School of Medicine at Mount Sinai, New York, NY USA

**Keywords:** Parkinson's disease, Gene expression, Computational biology and bioinformatics

## Abstract

Parkinson’s Disease (PD) is a debilitating neurodegenerative disorder, characterized by motor and cognitive impairments, that affects >1% of the population over the age of 60. The pathogenesis of PD is complex and remains largely unknown. Due to the cellular heterogeneity of the human brain and changes in cell type composition with disease progression, this complexity cannot be fully captured with bulk tissue studies. To address this, we generated single-nucleus RNA sequencing and whole-genome sequencing data from 100 postmortem cases and controls, carefully selected to represent the entire spectrum of PD neuropathological severity and diverse clinical symptoms. The single nucleus data were generated from five brain regions, capturing the subcortical and cortical spread of PD pathology. Rigorous preprocessing and quality control were applied to ensure data reliability. Committed to collaborative research and open science, this dataset is available on the AMP PD Knowledge Platform, offering researchers a valuable tool to explore the molecular bases of PD and accelerate advances in understanding and treating the disease.

## Background & Summary

Parkinson’s disease (PD) is a complex neurodegenerative disorder that significantly diminishes the quality of life of affected individuals by impairing motor skills and often impacting cognitive function. Neuropathological characteristics include the buildup of α-synuclein protein within neurons, leading to the formation of Lewy bodies and Lewy neurites^1,2^, alongside the degeneration of dopamine-producing neurons^3^. This condition manifests through symptoms such as tremors, stiffness, and memory loss, which progressively worsen, affecting daily activities and overall well-being^4^. The complex pathophysiological mechanisms that erode cognitive abilities in Parkinson's are still not fully understood. This knowledge gap highlights the need to examine changes in gene expression, which could reveal the underlying mechanisms of PD progression and enhance the potential for early diagnosis and the development of more effective treatments.

**Fig. 1:**
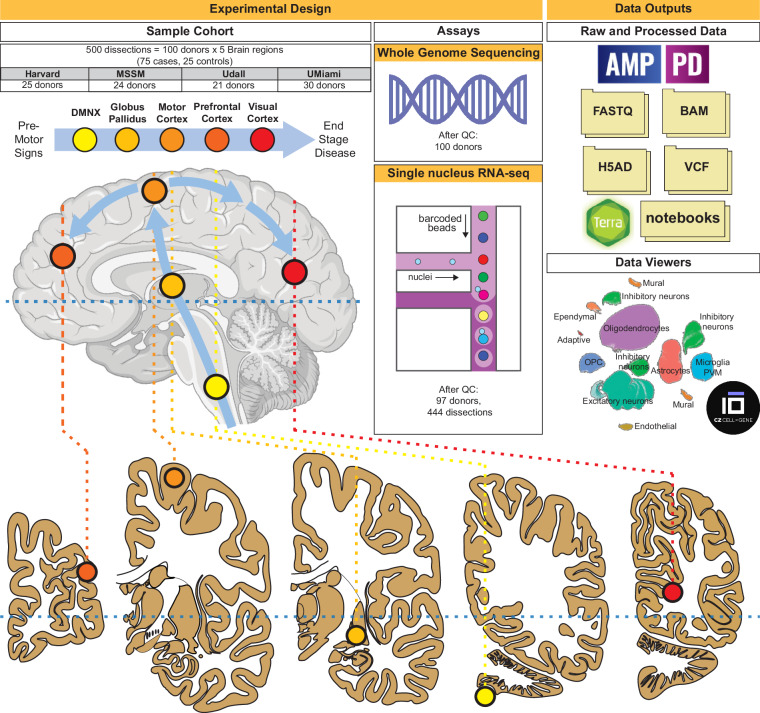
Schematic overview of dataset collection and main study deliverables. Harvard: NIH NeuroBiobank at the Harvard Brain Tissue Resource Center; MSSM: NIH NeuroBioBank at the Mount Sinai Brain Bank; Udall: University of Miami Udall Center of Excellence for Parkinson's Disease Research; UMiami: NIH NeuroBioBank at the University of Miami and University of Miami Brain Endowment Bank.

Alterations in gene expression and cell type composition are common disruptions in neurodegenerative disorders, including PD^5–10^. Traditional approaches^11^ such as bulk or cell-sorted tissue analyses have not been able to fully capture the complex molecular changes in PD, mainly due to the confounding effects of changes in cell composition^12^. This problem is worsened by the narrow focus of previous research, which has tended to concentrate on small sample sizes and specific brain areas, notably the substantia nigra, where there is a substantial decrease in dopaminergic neurons^3^. Moreover, the typical use of case-control studies, primarily involving patients in advanced stages of PD, fails to effectively track the gradual changes in gene expression that occur as the disease progresses. In our study, we have adopted several strategies to overcome these limitations: First, we utilized a single-nucleus RNA-seq assay (snRNA-seq) for data generation, enabling cell-specific analysis. Secondly, in collaboration with four different brain banks, we gathered extensive clinical and demographic records for over 600 PD cases. From this collection, we selected age- and sex- balanced specimens from 75 PD patients at varying disease stages, based on neuropathological evaluations and staging, and included 25 unaffected controls. To ensure coverage of all disease stages, we utilized Braak PD staging^13^, which quantifies regional disease progression and accumulation of Lewy bodies, primarily composed of α-synuclein. This approach enabled the inclusion of donors with early-stage PD pathology. Furthermore, we expanded the scope of transcriptomic profiling to include five brain regions beyond the extensively studied substantia nigra, tracking the progression of abnormal immunostaining α-synuclein patterns as defined by Braak PD stages^13^ (Fig. 1). This included early affected (DMNX: dorsal motor nucleus of the Xth nerve; GPI: globus pallidus interna) to late-affected regions (PMC: primary motor cortex, DLPFC: dorsolateral prefrontal cortex), as well as a largely unaffected region (PVC: primary visual cortex). The corresponding Brodmann areas (BA) for these regions are as follows: PMC = BA4, DLPFC = BA9 and PVC = BA17.

For whole genome sequencing (WGS), we utilized genomic DNA extracted from the PVC (Fig. 1). The data described in this study represent the largest PD-oriented single nucleus data collection to date and is available at the AMP PD Knowledge Platform.

## Methods

### Cohort data collection

**Fig. 2:**
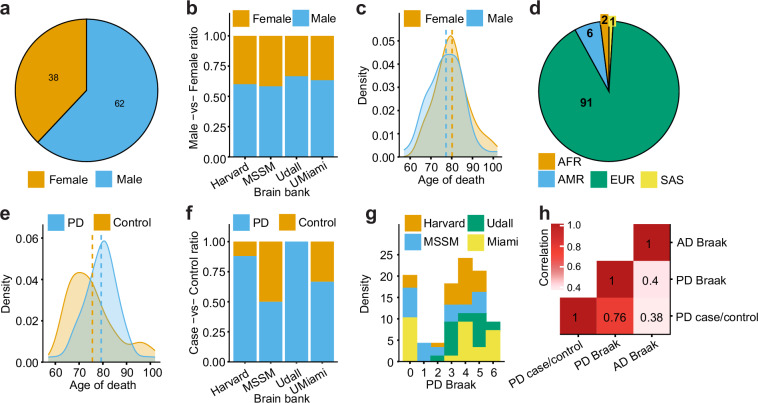
Study cohort characteristics. (**a**–**c**) Numbers and distributions of male and female donors across brain regions and age. (**d**) Distribution of donors by the most similar ancestry superpopulation predicted by QDA (AFR: African, AMR: Admixed American, EUR: European, SAS: South Asian). (**e**–**g**) Distribution of donors by age, source brain bank and Braak PD staging. (**h**) Spearman correlation coefficients among PD-related phenotypes. Harvard: NIH NeuroBiobank at the Harvard Brain Tissue Resource Center; MSSM: NIH NeuroBioBank at the Mount Sinai Brain Bank; Udall: University of Miami Udall Center of Excellence for Parkinson's Disease Research; UMiami: NIH NeuroBioBank at the University of Miami and University of Miami Brain Endowment Bank.

The cohort consists of genetic (WGS) and transcriptomic (snRNA-seq) assays collected using a cohort of 100 donors sourced from the following brain banks: NIH NeuroBioBank at the Mount Sinai School of Medicine, NIH NeuroBioBank at the Harvard Brain Tissue Resource Center, NIH NeuroBioBank at the University of Miami, the University of Miami Brain Endowment Bank and the University of Miami Udall Center of Excellence for Parkinson's Disease Research (Fig. 1 and Table 1). All data were obtained from biobanks with appropriate informed consent from all participants. Detailed cognitive, neuropathological, and demographic information was gathered for all donors, who were mainly of European descent with a male-to-female ratio of 3:2 (Fig. 2a–d). The study included 75 donors across a spectrum of PD severity, based on the Braak PD staging (Braak *et al*.^13^), which tracks the spread of Lewy body pathology (Fig. 2e–g), and 25 donors without the disease as control subjects (Table 1). Additionally, data were collected on Alzheimer’s disease (AD) Braak staging^14^, which evaluates tau neurofibrillary tangle accumulation, and the Hoehn and Yahr scale^15^, assessing functional disability in PD. The importance of examining both neuropathological (Braak PD staging) and detailed clinical characteristics of PD is evident from only a limited correlation observed between those phenotypes (Fig. 2h). Furthermore, the availability of both Braak AD and Braak PD stages provides an opportunity to explore the transcriptomics basis of symptomatic, clinical^16^, and, to a lesser extent, genetic overlaps^17,18^ between Tau and Lewy body accumulation. However, it is important to note that all donors were either unaffected controls or clinically diagnosed exclusively with PD but no other neurological or major neuropsychiatric diseases, including AD.

**Table 1:** Summary of clinical and demographics data stratified by source brain bank.

Brain Bank	#	Disease Status		Sex		Age			Data Available		
		Case	Control	Male	Female	<70	70–84	>85	Clinical	WGS	snRNAseq
UD^a^	21	21	0	14	7	1	13	7	21	21	21
HA^b^	25	22	3	15	10	6	15	4	25	25	25
UM^c^ NIH NeuroBioBank	17	12	5	10	7	3	9	5	17	17	15
UM^c^ Brain Endowment Bank	13	8	5	9	4	3	7	3	13	13	12
MS^d^	24	12	12	14	10	3	19	2	24	24	24
Total	100	75	25	62	38	16	63	21	100	100	97

^a^Udall Center of Excellence for Parkinson’s Disease Research (UD).

^b^NIH NeuroBioBank at Harvard Brain Tissue Resource Center (HA).

^c^University of Miami (UM).

^d^NIH NeuroBioBank at the Mount Sinai Brain Bank (MS).

### snRNA-seq data generation

#### Nuclei isolation and snRNA-seq library preparation

All buffers were supplemented with RNAse inhibitors (Takara). Six samples, each from a different individual, were processed in parallel. Twenty-five mg of frozen postmortem human brain tissue from each specimen was homogenized in cold lysis buffer (0.32 M Sucrose, 5 mM CaCl2, 3 mM Magnesium acetate, 0.1 mM, EDTA, 10 mM Tris-HCl, pH8, 1 mM DTT, 0.1% Triton X-100) and filtered through a 40 µm cell strainer. The flow-through was underlaid with sucrose solution (1.8 M Sucrose, 3 mM Magnesium acetate, 1 mM DTT, 10 mM Tris-HCl, pH8) and centrifuged at 107,000 g for 1 hour at 4°C. Pellets were resuspended in PBS and quantified (Countess II, Life Technologies). 2 million nuclei from each sample were then pelleted at 500 g for 5 minutes at 4˚C and re-suspended in 100 µl staining buffer (2% BSA, 0.02% Tween-20, 10 mM Tris, 146 mM NaCl, 1 mM CaCl_2_ and 21 mM MgCl_2_). Each sample was incubated with 1 µg of a distinct TotalSeq-A nuclear hashing antibody (Biolegend) for 30 min at 4°C. Prior to Fluorescence-Activated Nuclei Sorting (FANS), volumes were brought up to 250 µl with staining buffer and 7-AAD (Invitrogen) added to facilitate the detection of nuclei. 7-AAD positive nuclei were sorted into tubes pre-coated with 5% BSA using a FACSAria flow cytometer (BD Biosciences).

Following FANS, nuclei were washed in staining buffer before being re-suspended in 22 µl PBS and quantified. Nuclei concentrations were normalized and equal amounts from each sample were pooled together. Two aliquots of 60,000 pooled nuclei (i.e. 10,000 per sample) were processed in parallel using 3’ v3.1 reagents (10x Genomics). At the cDNA amplification step, reactions were supplemented with a hash-tag oligo (HTO) cDNA “additive” primer (GTGACTGGAGTTCAGACGTGTGCTCTTCCGAT*C*T; *Phosphorothioate bond). Following cDNA amplification, supernatants from the 0.6x SPRI selection step were retained for HTO library^15^ generation. Otherwise, cDNA libraries were prepared according to the manufacturer’s instructions (10x Genomics). HTO libraries were prepared as described previously^19^. All libraries were sequenced at the New York Genome Center (NYGC) using the Novaseq 6000 platform (Illumina).

#### Computational processing

Alignment of sequencing reads from each multiplexed sample batch was conducted using the STARsolo (v.2.7.9a)^20,21^ algorithm against the hg38 reference genome. To assign the cells from each sequencing pool to their respective donors, we used a genotype-based demultiplexing strategy followed by a genotype concordance check. Initially, cellSNP-lite (v.1.2.0)^22^ collected allele data from polymorphic loci overlapping snRNA-seq reads from genes expressed in at least 10 cells. These polymorphic loci were required to display a minor allele frequency of at least 0.1 and meet the UMI threshold of 20. Subsequently, the vireo (v.0.5.8)^23^ segregated cells into clusters that corresponded to the six distinct donors in each batch. Identity verification for each cell cluster was performed through a genotype concordance analysis using the QTLtools-mbv (v.1.3)^24^, comparing cell clusters against WGS. To ensure the accuracy of this procedure, we filtered out cells not meeting baseline quality control (QC) metrics, i.e. minimum number of expressed genes (n ≥ 1,000) and maximum fraction of mitochondrial reads (less than 5%). Despite most pools containing the expected donors, genotype concordance data was crucial for identifying and correcting sporadic instances of sample mislabeling or swapping.

Following alignment and donor assignment, a stringent, three-tiered QC protocol was employed to eliminate ambient RNA and ensure only viable cells were retained for subsequent analyses. Initially, a rigorous cell-level QC was implemented, which built on preliminary checks from the demultiplexing phase. Cells falling outside the defined ranges for UMI counts (1,500 to 110,000), gene expression (1,100 to 12,500 genes), and mitochondrial content (below 2%) were excluded. This stage also included assessment for potential ambient RNA contamination, particularly from non-messenger RNAs such as rRNA, sRNA, pseudogenes, and the lncRNA MALAT1. Additionally, cell doublets were identified and removed using the Scrublet (v.0.2.3)^25^. The second QC stage focused on gene expression, removing genes not consistently expressed in at least 0.05% of nuclei. The final QC step targeted the sample level, excluding samples represented by fewer than 50 cells to minimize noise in downstream analysis.

#### Cell clustering

All 2,232,626 nuclei resulting from the previous QC steps were unified into a single dataset. Normalization and clustering were performed using the SCANPY (v.1.9.3)^26^ and Pegasus package^27^. Briefly, counts for all nuclei were scaled by the total library size and logarithmically transformed. Subsequently, 6,000 highly variable genes were identified based on dispersion and mean (excluding sex chromosomes and mitochondria-related genes), followed by regression of the technical influence of the total number of counts, percentage of mitotic counts and cell cycle difference using *pg.regress_out()* function in Pegasus. Furthermore, the data were also corrected for batch effects coming from different brain banks using the Harmony^28^ approach via the *pg.run_harmony()* function. Principal Component Analysis (PCA) was carried out on the variable genes, followed by Uniform Manifold Approximation and Projection (UMAP)^29^ dimensionality reduction on the top 30 principal components (PCs). We confirmed that more than 30 PCs capture 100% of the data variance. The top 50 PCs were utilized to construct a k-nearest-neighbors cell–cell graph with k=100 neighbors. The Leiden algorithm was then applied to identify cell clusters. These analyses were performed using the functions *pg.pca()*, *pg.elbowplot()*, *pg.neighbours()* in Pegasus, and leiden clustering using *sc.tl.leiden()* in SCANPY. Differential gene expression analysis for each cluster was conducted using the variance-adjusted t-test implemented in the *sc.tl.rank_genes_groups()* function in SCANPY. The top 300 ranking genes for each cluster were extracted and tested for overlap with previously reported markers^30–32^. Subsequently, during iterative sub-clustering, additional potentially dubious clusters representing low-quality or doublet cells were identified based on extreme separation from the rest of the sub-cluster population from the same cell type. Among these, clusters characterized by a distinctly high number of total counts or/and mixed expression of markers from different cell types were detected as potential doublets and excluded from downstream analyses, resulting in a total of 2,096,155 nuclei retained. Furthermore, cellular identities at the class level of taxonomy were confirmed by examining cosine similarity correlations which compared to pseudo bulk-level transcriptome of detected Leiden clusters with reference datasets^30–32^.

### Whole-genome sequencing

#### Library preparation

DNA was extracted from tissue samples using the QIAmp DNA kit (Qiagen, kit number 51306), according to the manufacturer’s instructions. Once DNA was extracted, samples were quantified using the Qubit Fluorometer (Life Technologies) and PicoGreen (Thermo Fisher), and sample quality was evaluated by checking Fragment Analyzer (Advanced Analytical) traces. WGS libraries were prepared using the Truseq DNA PCR-free Library Preparation Kit (Illumina, kit number 20015965, lot numbers 20698565 and 20706057) in accordance with the manufacturer’s instructions. Briefly, 1 µg of DNA was sheared using a Covaris LE220 sonicator (adaptive focused acoustics). DNA fragments underwent bead-based size selection and were subsequently end-repaired, adenylated, and ligated to IDT for Illumina TruSeq DNA UD Indexes (kit 20040870, lot number 20704419). Final libraries were quantified using the Qubit Fluorometer (Life Technologies) or Spectromax M2 (Molecular Devices) and library size determined using a Fragment Analyzer (Advanced Analytical) or Agilent 2100 BioAnalyzer. Libraries were sequenced on an Illumina Novaseq 6000 sequencer using 2x150bp cycles and S4 reagent kit v1.5 (catalog number 20028312).

#### Computational processing

Sequencing reads were aligned using BWA-mem^33^ to the hg38 reference genome. WGS variant calling was performed according to the Genome Analysis Toolkit (GATK, v.3.9.0) best practice recommendations^34^. Briefly, sample-level nucleotide variants (SNVs) and insertions/deletions (indels) were called using the GATK’s HaplotypeCaller and GenotypeGVCFs tools. To refine and annotate variants, Variant Quality Score Recalibration (VQSR) was conducted within the GATK framework. Sample-level QC followed established described pipelines^35–37^ involving an assessment of relatedness, DNA contamination (by VerifyBamID, v.1.1.3)^38^, sample-level missingness (exclusion when > 0.05), and overall coverage (exclusion when < 25x). Furthermore, outlier samples were checked against various metrics, including the number of called SNVs and indels, insert size length, alignment mapping quality score, CRAM file size, transition/transversion (Ti/Tv) ratio, the ratio of novel variants to all variants, and the mapped reads to paired reads ratio as previously described^35–37^. Variant-level filtering eliminated variants with missingness > 0.10 and high heterozygosity levels (InbreedingCoeff < −0.8). Individual genotype calls with depth < 10 or genotype quality < 20 were set as missing. Analyses were restricted to biallelic variants only.

#### Ancestry estimation

Based on the success of Mahalanobis distance techniques in ancestry assignment^39,40^, we leveraged quadratic discriminant analysis (QDA) to assign ancestry using scikit-learn (v.1.14.4)^41^. For each sample, we identified the most similar genetic ancestry group among the 1000 Genomes Project’s five superpopulations^42^. First, unimputed genotypes were merged with GRCh38 v2a 1000 Genomes Project data^42^ using BCFtools 1.9). PCs of the merged genotypes were computed using PLINK (v.2.0) PCA after variant-level filtering, i.e. retaining SNVs with minor allele frequency ≥ 0.01, Hardy-Weinberg equilibrium p-value ≥ 10^−10^ and variant-level missingness ≤ 0.01, followed by linkage disequilibrium pruning (window size = 1,000 kb, step size = 10, R^2^ = 0.2). Forward selection was used to select PC1 to PC6 to train the QDA models with regularization parameter 5^−7^.

## Data Records

All data described herein are available for use by the research community and have been deposited in the AMP PD Knowledge Platform^43^ (https://app.terra.bio/#workspaces/amp-pd-public/AMP-PD-In-Terra; select “AMP PD Release 4”). The released dataset encompasses files for snRNA-seq and WGS. For snRNA-seq, the data includes raw multiplexed sequencing files (FASTQ), sample-level and combined gene expression profiles (h5ad), and sample-level metadata. For WGS, the dataset comprises sample-level aligned sequencing data (CRAM), genomic variants (gVCF), and a set of QC files produced by Picard (v.2.22.3) and GATK (v.3.9.0), including insert size metrics, duplication metrics, GC bias metrics, alignment summary metrics, and WGS-specific metrics. The dataset webpage includes several Terra notebooks in Python and R for data analysis in a cloud environment. Access to this data is governed by a Data Use Agreement that permits its use for approved research and educational purposes by registered and compliant users. Single-cell data can be further inspected at CELLxGENE (RRID:SCR_021059) portal (https://cellxgene.cziscience.com/collections/d5d0df8f-4eee-49d8-a221-a288f50a1590).

## Technical Validation

### snRNA-seq data quality control

**Fig. 3:**
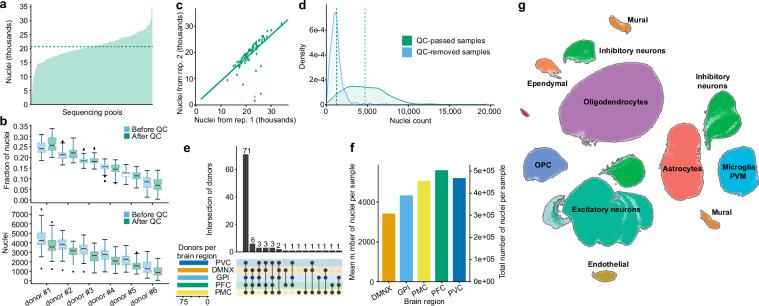
Analysis of snRNA-seq dataset. (**a**) Distribution of the number of nuclei across sequencing pools. Horizontal dashed line denotes a mean value. (**b**) Distribution of nuclei to replicates within pools, ordered by cell count. Each replicate is depicted using two boxplots representing the nuclei distribution before and after QC. The center line (black) indicates the median, the box shows the interquartile range, and the whiskers indicate the highest/lowest values within 1.5× the interquartile range. (**c**) Comparison of QC-passed nuclei counts between pairs of replicates from the same pools shows high consistency (Spearman’s *ρ*=0.79). (**d**) Distribution of nuclei counts in samples that passed or failed QC (vertical line indicates the mean values). (**e**) Sample counts and intersections among brain regions. (**f**) nuclei distribution across five brain regions. The left y-axis shows the average number of nuclei per sample for each region, while the right y-axis indicates the total number of nuclei detected in all samples from each region. (**g**) UMAP visualization of single nuclei defined by RNA-seq data shows eight major cell type clusters that are expected to be presented in the investigated brain regions.

After completing the QC process and excluding all low-quality samples, our snRNA-seq dataset included a total of 2,096,155 nuclei distributed across 161 pools. Each pool comprised six samples, and each pool was sequenced in replicate across two different flowcells. The average yield per pool was 20,640 nuclei (Fig. 3a) and these were expected to be evenly distributed across the samples. Despite this expectation, substantial variability in cell counts was observed, largely due to differences in the quality and condition of the samples within the same pool, which influenced cell viability and capture efficiency^44^. Typically, the largest sample captured about 25% of the nuclei (µ=3,680 nuclei), while the smallest captured about 7% (µ=1,043 nuclei) (Fig. 3b). Such variability is, however, not unusual and has been observed in other studies^23,44,45^. Despite these variations in per-sample nuclei count, the comparison of total number of nuclei between replicates showed almost perfect correlation (Spearman’s ρ=0.79), suggesting data robustness (Fig. 3c). In contrast, samples that were removed at the QC step contained approximately 70% fewer nuclei compared to those that passed QC (1,407 vs 4,721 cells, Fig. 3d). Donor representation across brain regions also varied: 71 donors were represented in each of the five brain regions and 18 were missing samples from only one region (Fig. 3e). Conversely, only one donor had samples from a single brain region and 3 donors did not generate any snRNA-seq data. Notably, regions affected early in Parkinson's disease exhibited significantly fewer cells than later affected, or putatively unaffected, regions (Fig. 3f). This suggests regional variations in cellular vulnerability and pathological progression, underscoring the importance of targeted studies to elucidate region-specific disease mechanisms in PD. Cell taxonomy identified nine major cell type clusters that are known to be present in the investigated brain regions (Fig. 3g).

### Whole-genome sequencing quality control

**Fig. 4:**
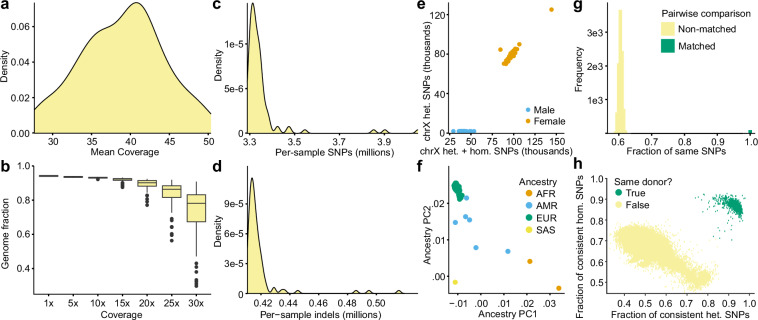
Quality control of WGS data. (**a**) Distribution of mean coverage indicating the average number of high-quality sequencing reads per base after applying all QC steps. (**b**) The fraction of the genome sequenced at different depths. The center line (black) indicates the median, the box shows the interquartile range, and the whiskers indicate the highest/lowest values within 1.5× the interquartile range. (**c,d**) Number of per-sample SNPs and indels. (**e**) Sex check based on comparison of the counts of heterozygous and homozygous alleles. (**f**) The first two PCs of genetic ancestry. (**g**) Pairwise comparison of the SNPs among all putatively matched and non-matched combinations of WGS samples. (**h**) Pairwise comparison of the genetic similarities calculated by QTLtools-mbv between WGS samples and genotypes called from snRNA-seq data.

The mean mapped coverage across all samples was 39x (±5x; Fig. 4a), with 94.2% (±0.3%) of the genome achieving at least 1x coverage and 93.1% (±0.3%) reaching at least 10x coverage (Fig. 4b). On average, each sample contained approximately 3.35 million SNPs (±0.15 million) and 418,813 indels (±15,274; Fig. 4c,d). Additionally, our analysis clearly distinguished male from female samples and demonstrated high concordance between inferred and self-reported ancestry across all donors (Fig. 4e,f). Pairwise genotype comparison between WGS samples as well as genotype comparison between WGS and snRNA-seq samples confirmed a clear separation between pairs from the same donors compared to those from different donors (Fig. 4g,h).

## Usage Notes

We would like to highlight the availability of additional clinical metadata beyond Braak PD staging and the binary clinical definition of PD case/control status. Specifically, we provide Braak AD staging data for 83% of the donors. Additionally, other metrics, including longitudinal data and detailed clinical features of PD, are available. These metrics use the Movement Disorder Society – Unified Parkinson’s Disease Rating Scale^46^, Mini-mental state examination^47^, Modified Schwab & England scale^48^, Epworth Sleepiness Scale^49^, and are primarily available for donors from UD (Table 2). The UD cohort consists of 21 donors, all diagnosed with Parkinson's disease and having a Braak PD stage of at least 2. Thus, the UD cohort does not cover the entire spectrum of disease progression, and users utilizing only data from UD should consider this limitation in their analyses.

**Table 2:** Summary of availability of clinical metadata stratified by source brain bank.

Table Name	Description	Brain Bank				Ref [PMID]
		UD	HA	UM	MS	
PD Medical History	initiation and use of PD medication, changes of diagnosis over time, indication of surgeries	X	X	X	X	
Family History PD	indication of PD diagnosis for father, mother and/or other relatives	X		X		
Smoking and alcohol history	indication and quantification of severity of smoking and alcohol consumption	X		X	X	
Epworth Sleepiness Scale	general level of daytime sleepiness	X				1798888
LBD_Cohort_Clinical_Data	clinical symptoms (e.g. visual hallucionations, loss of memory, mood disorder)	X	X	X	X	
LBD_Cohort_Path_Data	neuropathological assessments (CERAD^a^, AD Braak, PD Braak)	X	X	X	X	
MDS UPDRS^b^ Part I	non-motor symptoms covering aspects like mood, cognition and sleep	X				12815652
MDS UPDRS^b^ Part II	motor symptoms impacting daily activities	X		X		12815652
MDS UPDRS^b^ Part IV	motor complications related to PD disease treatment	X				12815652
MMSE^c^	screening for cognitive impairment and monitoring fo changes in mental status over time	X		X		1202204
Modified Schwab & England (ADL^d^)	assessment of the capabilities of people with impaired mobility	X				
UPDRS^b^	other UPDRS metrics not involved in Part I-IV	X				12815652

^a^CERAD: Consortium to Establish a Registry for Alzheimer’s Disease.

^b^MDS UPDRS: Movement Disorder Society – Unified Parkinson’s Disease Rating Scale.

^c^MMSE: Mini Mental State Examination.

^d^ADL: Activities of Daily Living.

Complete description of all metrics at https://amp-pd.org/harmonized-clinical-assessments.

Our study involves tissue samples from four brain banks, each contributing different proportions of cases and controls, males and females, and varying age-at-death distributions. To account for unwanted biological and technical variation, we recommend adjusting for relevant covariates from the metadata. The choice of covariates should align with the specific research question; for instance, sex should not be adjusted if studying sex-specific differences. In most of our studies, we typically account for demographic and technical factors such as sex, age, brain bank, RNA integrity number (RIN), and postmortem interval (PMI).

Access to the AMP PD Knowledge Platform data can be obtained by following the registration process outlined at http://amp-pd.org/register-for-amp-pd. This process includes the submission of a registration form, obtaining approval, and compliance with the AMP PD Data Use Agreement.

## Data Availability

The source code used to analyze the metadata and create figures for this manuscript can be found on GitHub at this location: https://github.com/DiseaseNeuroGenomics/AMP-PD_SciData. Additionally, jupyter notebooks for working with the dataset at Terra platform are available at this location: https://app.terra.bio/#workspaces/amp-pd-release-v4/Getting%20Started%20Tier%202%20-%20Clinical%20and%20Omics%20Access.
